# Serdexmethylphenidate/dexmethylphenidate effects on sleep in children with attention-deficit/hyperactivity disorder

**DOI:** 10.3389/fpsyt.2023.1193455

**Published:** 2023-06-23

**Authors:** Greg W. Mattingly, Ann C. Childress, Andrew J. Cutler, José Estrada, Meg Corliss

**Affiliations:** ^1^Washington University School of Medicine, Midwest Research Group, St. Louis, MO, United States; ^2^Center for Psychiatry and Behavioral Medicine, Las Vegas, NV, United States; ^3^SUNY Upstate Medical University, Syracuse, NY, United States; ^4^Neuroscience Education Institute, Lakewood Ranch, FL, United States; ^5^Corium, LLC, Boston, MA, United States

**Keywords:** ADHD, Azstarys, Children’s Sleep Habits Questionnaire, dexmethylphenidate, SDX/d-MPH, serdexmethylphenidate, sleep

## Abstract

**Introduction:**

Sleep-related problems are common in children with attention-deficit/hyperactivity disorder (ADHD). Sleep disorders are also side effects of all stimulant ADHD medications. Serdexmethylphenidate/dexmethylphenidate (SDX/d-MPH) is a once-daily treatment approved for patients age 6 years and older with ADHD. In this analysis, sleep behavior was assessed during SDX/d-MPH treatment in children with ADHD.

**Methods:**

In a 12-month, dose-optimized, open-label safety study in 6- to 12-year-old participants (NCT03460652), a secondary endpoint was assessment of sleep behavior based on the Children’s Sleep Habits Questionnaire (CSHQ) consisting of 8 sleep domains (bedtime resistance, sleep onset delay, sleep duration, sleep anxiety, night wakings, parasomnias, sleep-disordered breathing, and daytime sleepiness). This *post hoc* analysis examined the individual sleep domains in the 12-month safety study.

**Results:**

Of 282 participants enrolled, 238 were included in the sleep analysis. At baseline, mean (SD) CSHQ total sleep disturbance score was 53.4 (5.9). After 1 month of treatment, the mean (SD) CSHQ total score significantly decreased to 50.5 (5.4); least-squares mean change from baseline was −2.9 (95% CI: −3.5 to −2.4; *p* < 0.0001) and remained decreased up to 12 months. Mean sleep-score improvements from baseline to 12 months were statistically significant (*p* < 0.0001) for 5 of 8 sleep domains, including bedtime resistance, sleep anxiety, night wakings, parasomnias, and daytime sleepiness. Parasomnias and daytime sleepiness sleep domains showed the greatest mean improvement from baseline to 12 months. Sleep onset delay and sleep duration scores increased from baseline to 12 months. No statistically significant worsening occurred from baseline in sleep duration and sleep-disordered breathing domains; however, worsening of sleep onset delay was statistically significant.

**Conclusion:**

In this analysis of children taking SDX/d-MPH for ADHD, sleep problems did not worsen based on the mean CSHQ total sleep disturbance score. Statistically significant improvements in most CSHQ sleep domains were observed after 1 month and lasted for up to 12 months of treatment.

## 1. Introduction

Sleep-related problems are common in children with attention-deficit hyperactivity/disorder (ADHD) (1), and stimulant medications used for the treatment of children with ADHD can potentially exacerbate sleep problems (2). Insomnia is a common side effect of all stimulant ADHD medications (3). The effect of stimulant medications on sleep appears to vary depending on the patient’s health status, medication formulation, and dose of the stimulant treatment, as well as whether the sleep assessment was completed at the start of treatment or following extended use (3).

Methylphenidate (MPH) is the preferred and most widely used first-line treatment for children with ADHD because of its safety profile and its effects in reducing symptom severity (4, 5). Some studies have shown beneficial effects of MPH on sleep in patients with ADHD with pre-existing sleep disturbances (6, 7). Others have reported a more complicated relationship between MPH treatments and sleep in the ADHD patient population because the formulation and dose of MPH, length of treatment, measures of sleep (e.g., objective vs. subjective measures), and the patient population (e.g., inclusion of patients with sleep problems prior to treatment or those without any pre-existing sleep problems) can vary from study-to-study (6, 8).

Serdexmethylphenidate/dexmethylphenidate (SDX/d-MPH; Azstarys®) is a once-daily treatment approved for patients age 6 years and older with ADHD that contains a fixed molar ratio of 70% SDX and 30% d-MPH. SDX is a novel prodrug of d-MPH. The efficacy and safety results from a 1-month pivotal double-blind, laboratory classroom study of SDX/d-MPH in children age 6–12 years with ADHD were previously reported (9). The study showed that SDX/d-MPH treatment significantly improved ADHD symptoms compared with placebo (9). SDX/d-MPH had onset by 30 min postdose and up to 13 h duration of treatment effect (9). Its adverse events (AEs) profile was comparable with other stimulant treatments (9). No serious AEs were reported. During the dose optimization phase, 67% of participants reported AEs; the most common being insomnia and decreased appetite (9).

A subsequent 1-year, open-label, dose-optimized safety study of SDX/d-MPH showed that SDX/d-MPH was well tolerated with no new or unexpected safety findings and had sustained effectiveness in reducing ADHD symptoms during the 1-year treatment period in children age 6 to 12 years with ADHD (10). Of 238 participants assessed during the 12-month treatment phase of the study, the most common treatment-related AEs were decreased appetite (18.5%), upper respiratory tract infection (9.7%), nasopharyngitis (8.0%), decreased weight (7.6%), and irritability (6.7%). Notably, rates of insomnia were relatively low, as 5.3% of participants reported initial insomnia during the dose-optimization phase and 5.0% of participants reported insomnia during the treatment phase (10). The objective of this *post hoc* analysis was to assess individual sleep domains of bedtime resistance, sleep onset delay, sleep duration, sleep anxiety, night wakings, parasomnias, sleep-disordered breathing, and daytime sleepiness using the Children’s Sleep Habits Questionnaire (CSHQ) (11) in children treated for ADHD for up to 1 year with SDX/d-MPH.

## 2. Materials and methods

### 2.1. Study design

This 1-year, dose-optimized, open-label safety study analyzed SDX/d-MPH administered orally in children age 6–12 years with ADHD (NCT03460652). The study design was reported previously (10). The first participant was screened on February 21, 2018, and the last participant completed the study on June 27, 2019. The study consisted of a 30-day screening phase, a 3-week dose-optimization (DO) phase (for new participants), a 360-day treatment phase, and a follow-up visit. New participants were enrolled in the current study, and participants from the pivotal double-blind laboratory classroom study were rolled over into the current trial within 45 days of their last dose of SDX/d-MPH from the double-blind study (rollover participants).

For new participants only, during the DO phase, participants started treatment with 39.2/7.8 mg SDX/d-MPH daily for 7 days. Dose adjustments, if needed, were performed at approximately weekly intervals. The dose at the end of the third week was assigned as the optimized dose consisting of 26.1/5.2 mg, 39.2/7.8 mg, or 52.3/10.4 mg SDX/d-MPH daily, which are 20-, 30-, and 40-mg molar equivalent doses of total d-MPH HCl. For the new participants, the starting dose during the treatment phase was the optimized SDX/d-MPH dose at the end of the DO phase. For rollover participants, the starting dose in the treatment phase was the same as their optimized dose from the previous study.

The primary end point was safety and tolerability of SDX/d-MPH. The *post hoc* analysis was of sleep behavior based on the CSHQ assessments (11), which was a secondary end point (10).

Criteria for exclusion from this study have been described previously (10). New participants were excluded from the study if they had psychiatric comorbidities, including any diagnosis of bipolar I or II disorder, major depressive disorder, conduct disorder, or obsessive–compulsive disorder or any history of psychosis, autism spectrum disorder, disruptive mood dysregulation disorder, intellectual disability, Tourette syndrome, or confirmed genetic disorder with cognitive and/or behavioral disturbances. Participants with oppositional defiant disorder were permitted to enroll in the study if it was not the primary focus of treatment and, in the opinion of the investigator, was mild to moderate, and as long as eligible participants with oppositional defiant disorder were appropriate and cooperative during screening. Participants were also excluded if they had taken medications from more than one class of ADHD treatments within 30 days prior to the screening period. New participants had to wash out current stimulant ADHD medications, including herbal medications, from 5 days prior to the start of the DO phase, and abstain from taking these to the end of a follow-up visit or early termination; and wash out nonstimulant ADHD medications from 14 days prior to the start of the DO phase, and abstain from taking these to the end of the follow-up visit or early termination. Rollover participants had the same entry and exclusion criteria in the preceding double-blind study.

### 2.2. Sleep assessment

The CSHQ is a validated screening tool for the assessment of sleep habits in children (11) and is used worldwide for screening of sleep disturbances in children with ADHD and those treated with stimulants for ADHD (12–14). The CSHQ assesses 8 sleep domains: bedtime resistance, sleep onset delay, sleep duration, sleep anxiety, night wakings, parasomnias, sleep-disordered breathing, and daytime sleepiness (11). CSHQ scores were obtained during a clinician-directed interview with the parent/guardian/caregiver at the visits in the dose-optimization phase for new participants and in the treatment phase for all participants. For the *post hoc* analysis, CSHQ assessments and baseline data were analyzed using the treatment phase safety population, which included participants who received ≥1 dose of SDX/d-MPH in the treatment phase and had ≥1 postdose safety assessment in the treatment phase. Of a total CSHQ score of 99, the clinical cutoff indicating the presence of sleep disorders is a score ≥ 41 (11), and reductions in CSHQ scores indicate a trend toward improvement in sleep.

### 2.3. Statistical analyses

For each CSHQ sleep domain score and the total sleep disturbance score, a mixed model for repeated measures using restricted maximum likelihood was used to model the change from baseline, with baseline score included as a covariate in the model. Diagnostic plots were used to assess model assumptions. The significance of the change from baseline was tested for each domain. Bonferroni adjustments were made based on the number of CSHQ domains tested, and *p*-values were compared with *α* = 0.0056 (0.05/9) for significance testing. All statistical analyses were performed using SAS version 9.4.

## 3. Results

### 3.1. Participants

**Figure 1:**
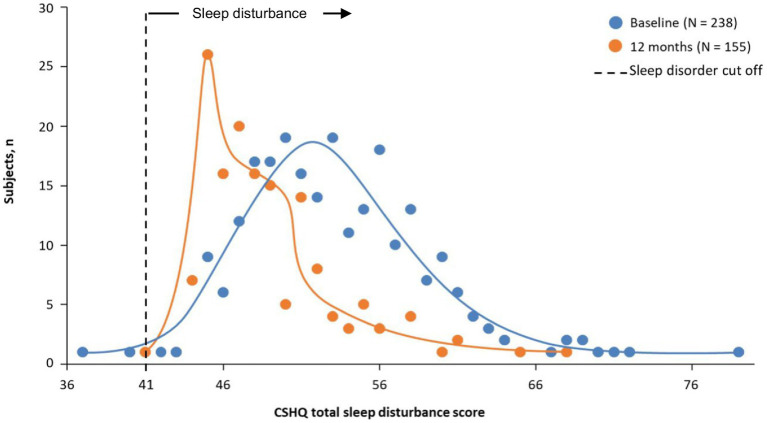
Frequency distribution of CSHQ total sleep disturbance score at baseline and 12 months. CSHQ, Children’s Sleep Habits Questionnaire. Treatment phase safety population.

Participant disposition was reported previously (10). Of 282 participants enrolled (212 new and 70 rollover), 238 were included in the sleep analysis. Participants were mostly male (60.9%), White (47.5%) and Black/African American (46.6%), and most participants reported sleep disturbances at baseline. The baseline mean (SD) CSHQ total score was 53.4 (5.9). Additional participants’ demographics and baseline characteristics are shown in Table 1. The distribution of the CSHQ scores at baseline is shown in Figure 1. Only 2 participants had a CSHQ score below 41 (scores of 37 and 40). The remaining 236 participants’ scores ranged from 42 to 79.

**Table 1:** Participant demographics and baseline characteristics. Treatment phase safety population.

Parameter	Participants
	(*N* = 238)
Age, mean (SD), y	9.1 (1.87)
Sex, *n* (%)	
Male	145 (60.9)
Female	93 (39.1)
Ethnicity, *n* (%)	
Hispanic or Latino	45 (18.9)
Not Hispanic or Latino	193 (81.1)
Race, *n* (%)	
White	113 (47.5)
Black/African American	111 (46.6)
Multiracial	9 (3.8)
Asian	2 (0.8)
Other	2 (0.8)
American Indian/Alaska Native	1 (0.4)
Weight, mean (SD), kg	38.6 (13.9)
Height, mean (SD), cm	139.6 (11.9)
Body mass index, mean (SD), kg/m^2^	19.3 (4.6)
CSHQ, mean (SD), total score	53.4 (5.9)
^1^ADHD-RS-5, mean (SD), overall score	41.5 (7.7)
^2^CGI-S, mean (SD), total score	4.7 (0.7)

ADHD-RS-5, Attention Deficit/Hyperactivity Disorder Rating Scale-5; CGI-S, Clinical Global Impressions-Severity; CSHQ, Children’s Sleep Habits Questionnaire.

1 ADHD-RS-5 measures the severity of ADHD symptoms on a scale from 0–54.

2 The CGI-S scale is a clinician-rated scale, that evaluates the severity of psychopathology (ADHD symptoms in the study) on a scale from 1 (not at all ill) to 7 (among the most extremely ill).

### 3.2. Sleep assessment

**Figure 2:**
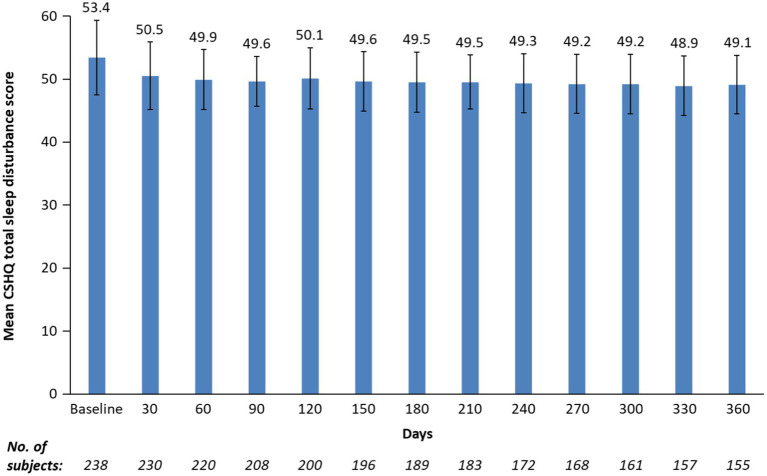
Mean CSHQ total sleep disturbance score by study visits for up to 12 months. CSHQ, Children’s Sleep Habits Questionnaire. Bars are standard deviations. Treatment phase safety population.

After 1 month of treatment, the mean (SD) CSHQ total sleep disturbance score significantly decreased from a baseline of 53.4 to 50.5 (5.4; Figure 2; least-squares mean change from baseline −2.9 [95% CI: −3.5 to −2.4; *p* < 0.0001]) and remained in the 48.9 to 50.1 range for up to 12 months, indicating sustained overall sleep improvement. Although total sleep disturbance scores were almost all ≥41 (99.2% baseline and 100% at 12 months), there was a shift towards a higher number of participants with lower total sleep disturbance scores at 12 months vs. at baseline (Figure 1).

Mean sleep-score improvements from baseline to 12 months were statistically significant (*p* < 0.0001) for 5 of 8 sleep domains. These included bedtime resistance, sleep anxiety, night wakings, parasomnias, and daytime sleepiness (Table 2). Parasomnias and daytime sleepiness sleep domains showed the greatest mean improvement from baseline to 12 months. Sleep onset delay and sleep duration scores increased from baseline to 12 months. There was no statistically significant worsening from baseline in sleep duration and sleep-disordered breathing domains; however, worsening of sleep onset delay was statistically significant (Table 2).

**Table 2:** Mean and change from baseline in CSHQ sleep domain scores. Treatment phase safety population.

Sleep domain	Baseline mean (SD)	SDX/d-MPH treatment at 12 months		
		Mean (SD)	LS mean change from baseline (95% CI)	*p* value
Bedtime resistance	10.8 (1.7)	10.1 (1.1)	−0.7 (−0.9, −0.6)	*p* < 0.0001
Sleep onset delay	2.2 (0.8)	2.6 (0.7)	0.4 (0.3, 0.5)	*p* < 0.0001
Sleep duration	6.6 (1.1)	6.8 (0.7)	0.2 (0.04, 0.28)	*p* = 0.0091
Sleep anxiety	5.5 (2.0)	4.6 (1.5)	−0.9 (−1.0, −0.7)	*p* < 0.0001
Night wakings	4.1 (1.3)	3.6 (1.0)	−0.5 (−0.6, −0.4)	*p* < 0.0001
Parasomnias	8.9 (1.9)	7.9 (1.4)	−1.1 (−1.3, −0.9)	*p* < 0.0001
Sleep-disordered breathing	3.4 (0.9)	3.2 (0.6)	−0.1 (−0.18, −0.02)	*p* = 0.0111
Daytime sleepiness	14.6 (3.02)	12.6 (2.8)	−2.0 (−2.4, −1.7)	*p* < 0.0001

LS, least-squares; SDX/d-MPH, serdexmethylphenidate/dexmethylphenidate.

## 4. Discussion

In this 1-year study of children taking SDX/d-MPH for ADHD, statistically significant improvements in most CSHQ sleep domains were observed after 1 month and lasted for up to 12 months of treatment. Nearly all participants entered the study with preexisting sleep disturbances as observed by a CSHQ score > 41. After 12 months of treatment with SDX/d-MPH, noticeably more participants had a lower sleep disturbance score from baseline; however, overall sleep remained in the category of clinical disturbances (overall score > 41). Importantly, these findings show that the use of SDX/d-MPH does not worsen CSHQ total score from baseline.

ADHD is associated with sleep disturbances independent of medication treatment, with one study reporting up to 73% of children with ADHD having some measure of sleep impairment (15). However, the measurement of sleep in the ADHD population is complicated as sleep problems are not only intrinsic to the disorder but can be caused or exacerbated by stimulant treatments (12). A 1-month randomized, double-blind, placebo-controlled study of 163 participants age 7 to 11 years with ADHD who were treatment naive found that of the children with parent-reported pre-existing sleep problems using the Pittsburgh Side Effects Rating Scale, a majority (62.5%) of the participants were deemed to no longer have sleep disturbances following treatment with the highest dose of MPH (6). However, participants without preexisting sleep problems did note some worsening of sleep with the highest dose of MPH. The findings suggested that children with ADHD who have preexisting sleep problems may experience beneficial sleep functioning effects with MPH treatment (6).

A cross-sectional survey of children age 4 to 10 years, excluding those with ADHD, which assessed sleep habits with the CSHQ, showed that the total score and subscale scores were consistently higher for patients referred to a pediatric sleep clinic vs. a control sample (age-matched participants in the community), thus validating the use of the CSHQ in assessing sleep in pediatric patients (11). Compared with patients referred to the pediatric sleep clinic (11), participants in the present study had higher baseline subscale scores in bedtime resistance (9.43 vs. 10.8), sleep onset delay (1.80 vs. 2.2), sleep duration (4.94 vs. 6.6), and daytime sleepiness (11.99 vs. 14.6). The higher scores for some sleep subdomains for participants in this study may be due to ADHD. Specific sleep disturbances known to occur in unmedicated children with ADHD include bedtime resistance, difficulty with morning awakenings, sleep onset difficulties, sleep-disordered breathing, night awakenings, and daytime sleepiness among other sleep problems (12). In the present study, mean sleep-score improvements from baseline to 12 months were statistically significant for 5 of 8 sleep domains, including bedtime resistance, sleep anxiety, night wakings, parasomnias, and daytime sleepiness. Sleep onset was negatively affected, and although sleep duration worsened, it was not significantly changed from baseline; however, improvements in the 5 sleep domains may have offset these two negatively affected sleep domains. Similar findings with sleep onset delay were reported previously with an extended-release formulation of MPH, assessed using the CSHQ (7). Two randomized, double-blind, placebo-controlled studies of an extended-release formulation of MPH (multilayer bead extended-release MPH [MPH-MLR]) administered to children with ADHD showed that MPH-MLR minimally affected sleep; however, not all CSHQ subscales of sleep improved. In the first study, during the 4-week open-label dose-optimization phase, statistically significant improvement from baseline was only observed for night wakings (7). Scores worsened for sleep onset delay and daytime sleepiness subscale scores, although the changes from baseline were not statistically significant (7). Sleep onset delay also worsened during the 2-week double-blind crossover phase. In the second study, during the 1-week double-blind phase, parasomnias significantly improved, but no significant differences were seen in the other subscales between MPH-MLR and placebo. At the end of the 11-week open-label, dose optimization phase, statistically significant improvements were seen in CSHQ total, bedtime resistance, sleep duration, night wakings, parasomnias, and daytime sleepiness. There was a worsening of sleep onset (7).

A limitation of this study is that nearly all participants reported sleep problems at baseline, which may have complicated this analysis of medication effect. Thus, the findings on sleep with SDX/d-MPH were mostly in participants with preexisting sleep disturbances. Additional study limitations include analyzing sleep behavior *post hoc* and not as the primary study objective, using only one subjective sleep assessment, using an open-label study design, lacking a control placebo or comparator product, and allowing some participants to discontinue the study over time, which may have led to selection bias in those who completed the entire 12 months.

## 5. Conclusion

In this study of children taking SDX/d-MPH for ADHD, sleep problems did not worsen based on the mean CSHQ total sleep disturbance score. Statistically significant improvements in most CSHQ sleep domains were observed after 1 month and lasted for up to 12 months of treatment.

## Data availability statement

The raw data supporting the conclusions of this article will be made available upon written request.

## Ethics statement

This study was reviewed and approved at the 18 study sites in the United States at which it was conducted. The protocol and amendments, informed consent form, and other information provided to the participants were approved by an institutional review board prior to each study center’s initiation. This study was conducted in accordance with the ethical principles originating from the Declaration of Helsinki and current Good Clinical Practices and in compliance with local regulatory requirements and Code of Federal Regulations. Written informed consent was obtained from all participants prior to enrollment into the study. At least 1 parent or legal guardian was required to provide written permission, and each participant had to give written or verbal permission for study participation. For verbal permission, the study procedure was documented and signed by a witness. A parent or guardian could not be the witness for a child’s verbal permission document.

## Author contributions

All the authors were actively involved in data interpretation, critical review of the data, article writing, and editing.

## Conflict of interest

GM discloses that in the last 3 years he received consultant fees or honoraria from AbbVie, Acadia, Alkermes, Avanir, Axsome, Boehringer Ingelheim, Eisai, Emalex, Ironshore, Intra-Cellular Therapies, Janssen, Lundbeck, Medgenics, Neos, Neurocrine, NLS-1 Pharma AG, Otsuka, Redax, Rhodes, Roche, Sage, Shire, Sunovion, Supernus, Takeda, Teva, and Tris Pharma. ACC discloses that she in the last 3 years acted as a consultant for Aadrvark, Arbor, Aytu, Ironshore, Neos Therapeutics, Neurocentria, Noven, Otsuka, Purdue, Rhodes, Sky, Sunovion, Tris Pharma, Zevra Therapeutics (previously KemPharm, Inc), Supernus, Jazz, Corium, LLC, and Lumos. She participated in speaker’s bureau for Takeda (Shire), Arbor, Ironshore, Neos Therapeutics, Tris Pharma, and Supernus; research support from Allergan, Takeda (Shire), Emalex, Akili, Arbor, Ironshore, Lumos, Neos Therapeutics, Otsuka, Purdue, Adlon, Rhodes, Sunovion, Tris Pharma, Zevra Therapeutics (previously KemPharm, Inc), Supernus, US Food and Drug Administration, and Servier. She received writing support from Takeda (Shire), Arbor, Ironshore, Neos Therapeutics, Purdue, Rhodes, Sunovion, and Tris Pharma; and advisory board for Takeda (Shire), Akili, Arbor, Cingulate, Ironshore, Neos Therapeutics, Neurovance, Otsuka, Purdue, Adlon, Rhodes, Sunovion, Tris Pharma, Supernus, NLS Pharma, and Corium, LLC. AJC discloses that in the last 3 years he acted as a consultant for AbbVie, Akili Interactive, Alkermes, Arbor Pharmaceuticals, Axsome, Boehringer Ingelheim, Corium, LLC, Intra-Cellular Therapies, Ironshore Pharmaceuticals, Neurocrine, NeuroSigma, Noven, Otsuka, Purdue Canada, Reviva Pharmaceuticals, Sunovion, Supernus, Shire/Takeda, Teva, and Tris Pharma. He participated in speaker’s bureau for AbbVie, Alkermes, Arbor Pharmaceuticals, Axsome, Corium, LLC, Intra-Cellular Therapies, Ironshore Pharmaceuticals, Neurocrine, Noven, Otsuka, Sunovion, Supernus, Shire/Takeda, Teva, and Tris Pharma. He received research support from AbbVie, Aevi Genomics, Akili Interactive, Alkermes, Allergan, Arbor Pharmaceuticals, Zevra Therapeutics (previously KemPharm, Inc), Ironshore, Otsuka, Purdue Canada, Rhodes, Shire, Sunovion, Supernus, Takeda and Tris Pharma. JE and MC are both employees of Corium, LLC. The authors declare that this study received funding from Zevra Therapeutics (previously KemPharm, Inc) and Corium, LLC. The funders had the following involvement in the study: Zevra Therapeutics was involved in the clinical research, study design, and data collection; Corium LLC was involved in the post hoc analysis and funding the preparation of the manuscript.

## Publisher’s note

All claims expressed in this article are solely those of the authors and do not necessarily represent those of their affiliated organizations, or those of the publisher, the editors and the reviewers. Any product that may be evaluated in this article, or claim that may be made by its manufacturer, is not guaranteed or endorsed by the publisher.
